# Negative effects of adverse childhood experiences and absence of positive childhood experiences on healthcare employees: survey findings built on 10 years of trauma-informed development

**DOI:** 10.3389/fpubh.2024.1494587

**Published:** 2025-01-06

**Authors:** Laneita Williamson, Stephanie S. Daniel, Jennifer Carter, Adam Ridenhour, Camila A. Pulgar, Yasmin Gay, Beata Debinski

**Affiliations:** ^1^18 Cairns Consulting, Thurmond, NC, United States; ^2^Atrium Health Wake Forest Baptist, Winston-Salem, NC, United States; ^3^Department of Family and Community Medicine, School of Medicine, Wake Forest University, Winston-Salem, NC, United States

**Keywords:** trauma-informed care, adverse childhood experiences, positive childhood experiences, workforce development, health systems

## Abstract

**Introduction:**

Existing data on how history of trauma and adversity affects healthcare professionals is limited. This study sought to describe the prevalence of Adverse Childhood Experiences (ACEs) and Positive Childhood Experiences (PCEs) and their association with present-day workplace and wellbeing outcomes among a sample of healthcare teammates overall, as well as specifically among nurses. The paper also describes local trauma-informed care initiatives that supported study feasibility.

**Methods:**

Cross-sectional online survey data were collected in conjunction with promoting hospital-wide trauma-informed care training opportunities on two campuses that are part of an academic health system. Scales and items assessed independent variables PCEs and ACEs, and dependent variables including burnout, compassion fatigue, organizational belonging, adult resilience, access to support, and workplace violence (WPV). Demographic data were not collected to limit identifiability and encourage participation. Multivariable, hierarchical models regressed categorized total ACEs (ref 0, 1–3, 4–10) and total PCEs (ref 6–7, 3–5, 0–2) together on dichotomized dependent variables. Sub-analyses also adjusted for whether the participant was a nurse or reported serving in a different role.

**Results:**

Participants included 349 clinical and non-clinical employees, of whom 61.1% had at least one reported ACE, but 24.9% reported 4–10 ACEs. 29.9% reported only having 3–5 PCEs in their childhood, while 23.2% reported 0–2 positive childhood experiences. Even when adjusting for ACEs, having 0–2 PCEs was associated with significantly reduced odds of getting needed emotional or social support (AOR = 0.14, 95% CI: 0.07, 0.29). Having 0–2 PCEs relative to 6–7 PCEs was also significantly associated with greater odds of past 2-week compassion fatigue, a lower resilience score, and decreased odds of reported organizational belonging measures. Adjusting for ACEs and PCEs, nurses reported lower resilience and higher workplace violence compared to all other participant roles combined.

**Discussion:**

Teammate history of adversity was widespread and having fewer PCEs was associated with poorer adult workplace outcomes. These findings point to the value of a trauma-informed approach in healthcare, which offers healthcare organizations a framework for recognizing how trauma experiences affect wellbeing and intersect with the healthcare system, as well as how to create environments that are supportive of patients, staff, and providers.

## Introduction

An ever-growing body of evidence—beginning with the landmark adverse childhood experiences (ACE) study of the 1990s led by Dr. Vincent Felitti and Dr. Robert Anda (1)—shows that adverse childhood experiences (ACEs) are key sources of trauma, meaning they have enduring negative effects. The adversities most studied include different types of abuse, neglect, and household dysfunction (2), which have a dose response risk to over 40 biomedical outcomes, with a multifold increase if a child has experienced four or more ACEs (1). Moreover, ACEs during formative periods of brain development contribute to decreased ability to manage stress, conflict, emotional regulation, and impulse control (1). More recent literature also points to the importance of understanding not only ACEs but also how Positive Childhood Experiences (PCEs)—often operationalized as various types of support and connection in childhood [e.g., (3)]—contribute to wellbeing in adulthood. A large U.S. national study found PCEs were independently associated with lower risks of poorer adult mental and physical health wellbeing (4). Similarly, a systematic review identified that “[m]ost studies found promotive effects of PCEs whereby higher PCEs were associated with more favorable outcomes even after accounting for childhood adversity” (5), p. 10.

ACEs and PCEs are an important area of needed attention for healthcare systems as not only have patients and communities experienced widespread adversity but so has the workforce. Furthermore, national data from the Behavioral Risk Factor Surveillance System (BRFSS) (6), which conducts health-related surveys collecting information on risk-factors, chronic conditions, preventative services, and treatment, has shown that among respondents with higher numbers of ACEs, lower education level and socioeconomic status (SES) were associated with greater risks for negative long-term health outcomes (7). This suggests that many people intersecting with the health system who have the greatest needs and poorest health outcomes are carrying histories of trauma. Looking specifically at prior limited studies of healthcare professionals, nurses have been found to have significantly higher rates of four or more ACEs than the general population (8), pointing to a possible need for attending to histories of adversity and trauma and recognizing where there may be present day effects among this profession. Childhood adversities in healthcare workers, and again particularly for nurses, have demonstrated a great impact on adult psychological distress, perceived support, coping, and response (9).

Understanding histories of adversity and needs of the workforce is beneficial for informing possible points of intervention to mitigate negative outcomes in healthcare settings, which can be highly stressed environments. The interactions among patients and healthcare providers with a history of ACEs may be a contributory factor, as their exchanges could result from a heightened physiological response to pain, fear, and challenging circumstances.

Persistent stress that remains untreated due to an inability to identify the causes of heightened stress reactions or needs and ability to take measures to mitigate their impact can lead to more severe outcomes. In extreme cases, this uncontrolled stress may result in workplace violence, occurring either from patients or family members toward team members, or among team members themselves. It can also be a significant contributing factor to organizational trauma, which manifests as the workforce experiencing reduced job satisfaction, increased susceptibility to compassion fatigue, and heightened team conflict (10).

Although there are many ways that ACEs and PCEs may intersect with present day experiences in the workplace for healthcare professionals, much of the literature assesses one or a few of these upstream factors or downstream outcomes at a time. There is a gap in the literature exploring both ACEs and PCEs simultaneously in healthcare settings, as well as in exploring connections between multiple areas discussed so far, such as individual trauma, organizational context, resilience and managing stress, and workplace violence. As such, the primary objectives of this study were to describe the prevalence of ACEs and PCEs and their association with present-day workplace and wellbeing outcomes among a sample of healthcare teammates overall, as well as specifically among nurses. This research was made possible by a decade of nurse-led work preceding it and by concurrent efforts to deliver trauma-informed education, thus a secondary objective of this paper is to describe those efforts to build institutional awareness and support for pursuing a trauma-informed approach to healthcare and discuss how the empirical findings feed back into that work.

## Materials and methods

### Study context: delivering foundational trauma-informed care training

The opportunity to conduct this research relied upon authentic leadership support for trauma-informed care demonstrated in a way that would help encourage healthcare teammates to participate. This work was borne out of the first author’s experience that many healthcare professionals lacked knowledge of the neurological, biological, psychological, and/or social outcomes of individuals resulting from ACEs (11), which continues to be documented and observed to-date (9, 12, 13). Built on growing knowledge of the role of adversity in affecting future wellbeing and understanding of how individuals with trauma experiences intersect with service delivery systems, the United States Substance Abuse and Mental Health Services Administration (SAMHSA)—which chairs the National Interagency Task Force on Trauma Informed Care—was at the forefront of a national effort to develop a shared and widely applicable concept of trauma and propose a trauma-informed framework to guide organizational change (14). This framework advocates for adoption of six key principles [(1) safety; (2) trust and transparency; (3) peer support; (4) collaboration and mutuality; (5) empowerment, voice, and choice; and (6) cultural, historical, and gender issues] across 10 organizational domains to advance a trauma-informed approach. We used these principles in part to inform outcomes assessed in this study, described in greater detail below.

In 2012, legacy [Wake Forest Baptist Health] comprised a single academic center in [Winston-Salem, North Carolina, USA] (a small city in the central-west area of the state) affiliated with [Wake Forest University School of Medicine]. In this year, nurse champion [Laneita Williamson] BSN (Bachelor of Science in Nursing), created a trauma-informed care foundational training out of her expertise in neuroscience, and as an adjunct to her clinical duties. The new training was developed to respond to the growing gap between the developing knowledge base in the long-term effects of adversity and trauma, and healthcare employees’ awareness of it. It since evolved to provide a foundation in trauma and adversity and the impact on health and behaviors, teach about stress and resilience and how this applies to healthcare workers, as well as introduce trauma-informed care and its potential benefits at the organizational level to participants. The training was approved for delivery to nursing staff by the Nursing Professional Development Specialist (NPDS) and Education Department, and subsequently the nursing senate, and was with time evaluated for and granted continuing medical education credits.

Since 2013, [LW] had in turn led trainings in multiple medical and community specialty areas, tailoring content for varied clinical and non-clinical audiences, including clinical learners, chaplains, mental health providers, human resources, and local social service agencies and non-profit organizations (see Supplementary material for a full list of audiences trained to-date). *Ad-hoc* requests through word of mouth, slowly evolved into standing training for new nurses and clinical learners and grew to 100+ annual requests for the 90-min training by 2020, spanning four additional regional hospitals that had been added to the system (of which two served as the settings for this study).

#### Trauma-informed care task force

Broadening interest from 7 years of training contributed to the launch of the institutional Trauma-Informed Care Task Force, which convened an interdisciplinary team that included mental health clinicians, administrators, and researchers from different departments. The Task Force developed a guiding logic model for organizational change toward trauma-informed care and for associated research. Training demand surged following a merger with another health system (accompanied by an institutional name change to [Atrium Health Wake Forest Baptist]) and was compounded by the stress and adversity brought about by the COVID-19 pandemic. This increased interest was further bolstered by attention and dedicated support from the senior health system leadership, who endorsed expanded training reach. This, in turn, yielded the opportunity to scale training and conduct parallel data collection at the hospital campus level.

### Setting and training process

In 2022, one of the five hospital campuses in the area, located in a suburban setting, began a collaboration in partnership with the Trauma-Informed Care Task Force to launch a research study in conjunction with campus-wide availability of the foundational 90-min trauma-informed care training. To the best of the team’s knowledge, no other health systems in central-west [North Carolina] had implemented systematic, formalized training in trauma-informed care and conducted associated research to evaluate both workplace needs and effect of trainings or other interventions.

We conducted a research project that was promoted by senior executive leaders on the first campus (“Campus A”) as a pilot site for innovation in the system to help drive and test activities for foundational trauma-informed healthcare delivery and operational process improvements. The initiative was led on the ground by two local champions—authors Nurse Manager [Jennifer Carter], MSN (Master of Science in Nursing), RN (Registered Nurse) and Chaplain Dr. [Adam Ridenhour], who took on responsibility for coordinating efforts between the campus, teammates, leaders, and ancillary departments, and the Task Force, and who participated in developing the formative baseline assessment. The champions also enabled training delivery and data collection by connecting with the campus nurse educator; helping prepare materials for electronic registration and disclosures with the assessment, which included invitation and access information to the survey; collaborating with leadership on campus-wide email announcements announcing both the training and data collection; directly encouraging participation in both to teammates; and attending every training to assist with logistics and support participating teammate needs. Throughout, they monitored campus needs and continued to keep senior leadership appraised of progress and helped to present and share survey results to leadership at the conclusion of data collection. Inspired by efforts at the pilot site, a second campus location in our health system (“Campus B”)—located in a rural county—similarly launched preparation for campus-wide training and assessment in summer of 2023. The pilot site champions coordinated meetings with the second campus champion (also a nurse educator) and provided a warm hand-off on lessons learned and best practices.

### Data collection

The Task Force team developed a baseline formative survey to pilot with all teammates on both campuses. The survey was fielded through the Research Electronic Data Capture (REDCap) secure data collection and management system (15). All data collection was reviewed and approved by the [Wake Forest University School of Medicine] Institutional Review Board (exempt study IRB00091521).

Teammates campus-wide (clinic and non-clinical) were initially invited to participate in the survey through a launch email sent from the hospital president (shared across the two campuses), containing a general REDCap link for anonymous data collection. A subsequent reminder was sent from the hospital president several weeks later at each location, but the survey was also promoted by nurse managers and other team leaders, nurse educators, and by posting the survey QR code around campus. A final recruitment attempt was made by posting the survey QR code at the top of the training presentation to encourage participation by those who came in early and were waiting for the training to begin. It was advertised as taking about 10 min to complete. The link took potential participants to a landing page describing the research study; teammates gave their consent by clicking the button at the end of the page to continue to the survey. The survey for Campus A was in the field February—April 2023 (with added one-time data collection in June 2023 alongside a department-specific training request for a group that had been unable to attend an earlier offering), and for Campus B between September and November 2023.

### Measures

The larger survey included varying questions around topic areas including: (a) knowledge, attitudes, and practices of trauma-informed care; (b) participants’ primary role and years of experience; (c) caregiving responsibilities outside of work; (d) perceptions and experiences of workplace violence; (e) perceptions of the organizational context regarding workplace violence, and suggestions on how to make the work environment safer; (f) burnout, compassion fatigue, and organizational belonging; resilience and sources of support; (h) positive and adverse childhood experiences. Analyses for this study used the measures described below, including two independent variables and eight dependent outcome variables.

#### Participant characteristics

Due to the sensitive nature of many questions, as well as nursing team members’ experience that many staff do not complete or respond transparently to surveys launched through the institution due to fear of identifiability, we limited the number of participant characteristics that we collected. We did, however, ask whether the participant had received prior training in trauma-informed practice (with the following response options: yes, more than once before, yes, once before, and no); during a typical week how much of their time they spent on the study campus (50% or more/less than 50%); their primary role (categorized as administrative/nursing/physician or advanced practice provider (APP)/clinical, non-nursing, non-physician/non-clinical staff/other), their number of years of experience in the current role (<2 years/2–5 years/6–10 years/>10 years); the number of years of experience in healthcare (<5 years/5–10 years/11–15 years/16+ years); and whether they served in a leadership or decision-making role in their department, division, or unit (yes/no). To assess caregiving responsibilities outside of the workplace, we asked whether the respondent was a parent or caregiver for a child under the age of 18 (no/yes), and if they currently provided or had provided in the last year unpaid caregiving help to another adult (no/yes) (28).

#### Adverse childhood experiences (ACEs)

We used 10 ACEs measures from the BRFSS survey ACE module with minor modifications (6). To help encourage participation and promote privacy, we asked about ACEs in three sets (household dysfunction, neglect, and abuse), listed out each of the five, two, and three items respectively, then asked participants to write-in the number from each list that they had experienced prior to 18 years of age. We totaled the three subset scores to generate a total ACEs score ranging from 0 to 10, and subsequently created a categorical independent variable of 0 ACEs, 1–3 ACEs, 4–10 ACEs.

#### Positive childhood experiences (PCEs)

We used the seven PCEs items published by Bethell et al. (3), asking about various elements of childhood perceived support and connectedness, each rated on a 5-point Likert scale (never to very often). Also following analytical procedures published by Bethell et al. (3) we condensed responses for each PCE by grouping “very often” and “often” (yes) and “sometimes,” “rarely,” and “never” (no), and generated a score by totaling all ‘yes’ PCEs. We subsequently created a categorical independent variable, reverse coded as 6–7 PCEs, 3–5 PCEs, and 0–2 PCEs (3).

#### Burnout and compassion fatigue

We used two subsets of items from the Physician Fulfillment Index (16) to assess burnout and compassion fatigue as two different outcomes. Each asked to what degree the participant had certain experiences in the prior 2 weeks, with responses rated on a 5-point Likert scale and scored 0 to 4 (not at all [often] to extremely [often]). Four burnout items asked about feelings of a sense of dread, lack of enthusiasm, and physical and emotional exhaustion. Six compassion fatigue items asked about lower empathy with patients and with colleagues, lower sensitivity to others, less interest in talking with patients, and lower connectedness with patients and with colleagues due to work. Scores were totaled for each construct then divided by the number of items, and a cutoff point of 1.33 used to dichotomize participants into experiencing burnout or not (<1.33 average score) and experiencing compassion fatigue or not.

#### Adult support and resilience

We used a single item (3) to assess availability of support in adulthood (*“How often do you get the social and emotional support you need?”*) with responses on a 5-point Likert scale from ‘Always’ to ‘Never.’ Responses were dichotomized into Always/Usually getting needed support vs. Sometimes/Rarely/Never (3). We measured resilience using the 4-item Brief Resilient Coping Scale, that asked participants to rate responses on a scale of 1–5 to describe frequency of their coping behaviors (17). We added all item scores together (possible range 4–20) to generate a continuous resilience outcome variable, with a higher score indicating greater resilience.

#### Organizational belonging

Our study team developed two items to measure elements of organizational belonging and integrated some of the six trauma-informed principles. The first asked for agreement with the statement: “*I feel I am a valued, equal member within the department team and my voice is empowered to be collaborative in all areas, from patient care to processes,*” and the second with the statement “*I feel I can be autonomous and function to my highest capacity due to the safety, trustworthiness and transparency of the department in which I work.”* 5-point Likert scale responses ranged from ‘Not at all true’ to ‘Completely true,’ and were dichotomized for each into very/completely true vs. not at all/somewhat/moderately true.

#### Workplace violence (WPV)

We used two measures to assess experiences of WPV. The first was generated from the item: “*In the past 6 months, have you experienced any workplace violence, whether physical or non-physical?*” adapted from Copeland et al. (18). The outcome measure was reported as yes/no. Subsequent questions asked respondents to identify the person who used violence against them but are not reported here. The second item was developed by our team to capture organizational violence, asking: “*In the past 6 months, have you experienced any actions by your leadership or by anyone in a higher position of authority that you perceived as abusing their power? (e.g., intimidation, manipulation, chronic dismissal of your contribution, ongoing disrespect).*” Possible responses were yes/no.

### Analyses

All analyses were conducted in StataBE version 17 (StataCorp, College Station, TX, USA). We summarized participant characteristics descriptively overall, and by hospital campus. We also summarized distribution of our two independent and eight dependent variables, again overall and by hospital campus. Comparisons of response distributions between campuses were conducted using Fisher exact tests to assess for differences between locations. We subsequently assessed the distribution of reported ACEs and PCEs by participant characteristic, also using Fisher exact tests.

For analyses of the eight outcomes, we first conducted bivariate, multilevel regression using *gllamm*, clustered on campus, initially only looking at associations between outcomes and ACEs and between outcomes and PCEs. In turn, we conducted multivariate, multilevel regression again clustered on campus, but including both ACEs and PCEs as covariates. This was done to account for both positive and adverse experiences at the same time, and we used a hierarchical model to account for potential differences among the communities and workplace settings represented by the two hospitals.

Finally, to explore the experiences of nurses in greater depth, we first conducted sub-analyses focusing on nurses only: we again summarized distribution of our two independent and eight dependent variables, overall and by hospital campus. Subsequently, we dichotomized respondent role into “nursing” or “all others” and conducted multivariate, multilevel regression again clustered on campus including ACEs, PCEs, and dichotomized role categories as covariates.

## Results

Characteristics of 349 participants are presented in Table 1. Nursing staff were the largest group represented (53% overall) with a distribution of participants across role types; physicians or APPs were the least represented (2%). Respondents were experienced in healthcare; more than half (52%) had been in their current role for more than 10 years, and nearly 60% of participants had been in healthcare for 16+ years. Fewer than a third were in a leadership or decision-making role, though Campus A had a higher percentage of decision-makers participate—the only difference in participant characteristics between locations. Many had caregiving responsibilities for a child (44%) and/or an adult (42%) outside of their job roles.

**Table 1:** Sample characteristics, overall and by site; *p*-value from Fisher exact test.

Characteristic	Overall	Campus A	Campus B	*p*-value
	# (%)*	# (%)*	# (%)*	
Total number of participants	349 (100.0)	156 (100.0)	193 (100.0)	
Received prior training Trauma Informed Practice				0.81
Yes, more than once before	11 (3.2)	5 (3.2)	6 (3.1)	
Yes, once before	29 (8.3)	14 (9.0)	15 (7.8)	
No	308 (88.3)	136 (87.2)	167 (89.1)	
During typical week, time spent on study campus				0.86
50% or more	295 (84.5)	134 (85.9)	157 (83.4)	
Less than 50%	51 (14.6)	21 (13.5)	29 (15.5)	
Primary role				0.05
Administrative	27 (7.7)	17 (10.9)	10 (5.2)	
Nursing	184 (52.7)	75 (48.1)	109 (56.5)	
Physician/APP	8 (2.3)	7 (4.5)	1 (0.5)	
Clinical, non-nursing, non-physician	46 (23.8)	38 (24.4)	46 (23.8)	
Non-clinical staff	21 (10.9)	16 (10.3)	21 (10.9)	
Number of years of experience in current role				0.42
<2 years	46 (13.2)	25 (16.0)	20 (10.6)	
2–5 years	70 (20.1)	29 (18.6)	41 (21.8)	
6–10 years	50 (14.3)	25 (16.0)	25 (13.3)	
>10 years	182 (52.2)	77 (49.4)	101 (53.7)	
Number of years of experience in healthcare				0.75
<5 years	38 (10.9)	16 (10.3)	22 (11.7)	
5–10 years	55 (15.8)	24 (15.4)	29 (15.4)	
11–15 years	47 (13.5)	24 (15.4)	23 (12.2)	
16+ years	207 (59.3)	92 (59.0)	112 (59.6)	
In leadership or decision-making role in department/division/unit				**0.01**
No	242 (69.3)	101 (64.7)	137 (72.9)	
Yes	101 (28.9)	55 (35.3)	45 (23.9)	
Parent or caregiver for a child under the age of 18				0.11
No	191 (54.7)	81 (52.9)	108 (57.5)	
Yes	155 (44.4)	72 (47.1)	80 (42.5)	
Currently provide, or have provided in the last year, unpaid caregiving help to another adult				0.83
No	201 (57.6)	91 (58.3)	108 (57.5)	
Yes	148 (42.4)	65 (41.7)	80 (42.5)	

*Row percentages are rounded and may not total 100. Bold values indicate *p* < 0.05.

Nearly a quarter (24.9%) of respondents reported 4 or more ACEs, and 23.2% reported only 0–2 PCEs (Table 2). Burnout was nearly twice as common (43.3%) as compassion fatigue (22.3%) in the preceding 2 weeks, and 42.4% of participants reported they never, rarely, or only sometimes got the emotional or social support that they needed. As indicators of organizational belonging, more respondents endorsed having a sense of being able to work autonomously and function to highest capacity (53.8%) than for feeling like a valued, equaled, and empowered member of their team (43.7%). About one-third (32.3%) of participants reported experiencing any kind of WPV in the previous 6 months, and close to one-fifth (18.3%) reported experience any actions that reflected abuses of power in the organization in the previous 6 months. Responses on the two campuses differed significantly on three variables—getting needed emotional or social support, resilience, and feeling like a valued and empowered member of the team—all reflecting more positive experiences on Campus A.

**Table 2:** Distribution of responses for key variables, overall and by campus.

	Overall	Campus A	Campus B	*p*-value*
	*n* (%)	*n* (%)	*n* (%)	
Total	349 (100.0)	156 (100.0)	193 (100.0)	
Adverse childhood experiences (ACEs)				0.52
0 ACEs	133 (38.9)	57 (36.8)	76 (40.6)	
1–3 ACEs	124 (36.3)	55 (35.5)	69 (36.9)	
4–10 ACEs	85 (24.9)	42 (27.7)	42 (22.5)	
Positive childhood experiences (PCEs)				0.12
6–7 PCEs	160 (46.9)	65 (41.7)	95 (51.4)	
3–5 PCEs	102 (29.9)	48 (30.8)	54 (29.2)	
0–2 PCEs	79 (23.2)	43 (27.6)	36 (19.5)	
Burnout in past 2 weeks				0.39
No	198 (56.7)	93 (59.6)	105 (54.4)	
Yes	151 (43.3)	63 (40.4)	88 (45.6)	
Compassion fatigue in past 2 weeks				0.80
No	271 (77.7)	120 (76.9)	151 (78.2)	
Yes	78 (22.3)	36 (23.1)	42 (21.8)	
Frequency of getting needed emotional or social support				**0.005**
Never/rarely/sometimes	148 (42.4)	53 (34.0)	95 (49.2)	
Usually/always	201 (57.6)	103 (66.0)	98 (50.8)	
Resilience, *n* (mean, SE)	341 (14.7, 0.22)	152 (15.1, 0.21)	189 (14.3, 0.15)	0.009**
Organizational belonging: Feel a valued, equaled member of department/team and voice is empowered				**0.002**
Not at all/somewhat/moderately true	196 (56.3)	73 (47.1)	123 (63.7)	
Very/completely true	152 (43.7)	82 (52.9)	70 (36.3)	
Organizational belonging: Can be autonomous and function to highest capacity				0.10
Not at all/somewhat/moderately true	158 (46.2)	63 (41.2)	95 (50.3)	
Very/completely true	184 (53.8)	90 (58.8)	94 (49.7)	
Experienced any workplace violence (physical or non-physical), past 6 months				1.00
No	236 (67.8)	105 (67.7)	131 (67.9)	
Yes	112 (32.2)	50 (32.3)	62 (32.1)	
Experienced any actions by someone in position of authority perceived as abusing their power, past 6 months				0.78
No	282 (81.7)	128 (82.6)	154 (81.0)	
Yes	63 (18.3)	27 (17.4)	36 (19.0)	

*p-value comes from Fisher exact test.

**p-value comes from two-sided, two sample test of means (t-test in Stata).

Bold values indicate *p* < 0.05.

There were few significant associations between participant characteristics and categorized scores of ACEs (Table 3) or of PCEs (Table 4) for the full sample. Only the number of years of experience in the current role and in healthcare were associated with number of ACEs—appearing to show younger or less experienced teammates reported more ACEs—as well as caregiving for another adult outside of the workplace.

**Table 3:** Participant characteristics associated with ACEs, full sample.

	Total *n* (%)	Adverse childhood experiences			*p*-value*
		0 ACEs	1–3 ACEs	4+ ACEs	
		*n* (row %)	*n* (row %)	*n* (row %)	
Total score	349 (100.0)	133 (38.9)	124 (36.3)	85 (24.9)	
During typical week, time spent on study campus					0.25
50% or more	295 (84.5)	111 (38.4)	102 (35.3)	76 (26.3)	
Less than 50%	51 (14.6)	20 (40.0)	22 (44.0)	8 (16.0)	
Primary role					0.25
Administrative	27 (7.7)	11 (40.7)	7 (25.9)	9 (33.3)	
Nursing	184 (52.7)	64 (35.6)	66 (36.7)	50 (27.8)	
Physician/APP	8 (2.3)	6 (75.0)	2 (25.0)	0 (0.0)	
Clinical, non-nursing, non-physician	46 (23.8)	24 (40.5)	30 (35.7)	20 (23.8)	
Non-clinical staff	21 (10.9)	14 (38.9)	17 (47.2)	5 (14.0)	
Number of years of experience in current role					**0.001**
<2 years	46 (13.2)	12 (26.1)	16 (34.8)	18 (39.1)	
2–5 years	70 (20.1)	16 (24.2)	28 (42.4)	22 (33.3)	
6–10 years	50 (14.3)	26 (52.0)	12 (24.0)	12 (24.0)	
>10 years	182 (52.2)	79 (44.1)	68 (38.0)	32 (17.9)	
Number of years of experience in healthcare					**0.03**
<5 years	38 (10.9)	9 (23.7)	14 (36.8)	15 (39.5)	
5–10 years	55 (15.8)	18 (34.6)	16 (30.8)	18 (34.6)	
11–15 years	47 (13.5)	15 (32.6)	22 (47.8)	9 (19.6)	
16+ years	207 (59.3)	91 (44.4)	72 (35.1)	42 (20.5)	
In leadership or decision-making role in department/division/unit					0.67
No	242 (69.3)	90 (38.3)	83 (35.3)	62 (26.4)	
Yes	101 (28.9)	40 (39.6)	39 (38.6)	22 (21.8)	
Parent or caregiver for a child under the age of 18					0.06
No	191 (54.7)	82 (43.6)	67 (35.6)	39 (20.7)	
Yes	155 (44.4)	49 (32.5)	57 (37.8)	45 (29.8)	
Currently provide, or have provided in the last year, unpaid caregiving help to another adult					**0.04**
No	201 (57.6)	87 (44.4)	67 (34.2)	42 (21.4)	
Yes	148 (42.4)	46 (31.5)	57 (39.0)	43 (29.5)	

*p-value comes from Fisher exact test.

*Row percentages are rounded and may not total 100. Bold values indicate *p* < 0.05.

**Table 4:** Participant characteristics associated with PCEs, full sample.

	Total *n* (%)	Positive childhood experiences			*p*-value*
		0–2 PCEs	3–5 PCEs	6–7 PCEs	
		*n* (row %)	*n* (row %)	*n* (row %)	
Total score	349 (100.0)	160 (46.9)	102 (29.9)	79 (23.2)	
During typical week, time spent on study campus					0.50
50% or more	295 (84.5)	67 (23.3)	83 (28.8)	138 (47.9)	
Less than 50%	51 (14.6)	12 (24.0)	18 (36.0)	20 (40.0)	
Primary role					0.73
Administrative	27 (7.7)	7 (25.9)	5 (18.5)	15 (55.6)	
Nursing	184 (52.7)	44 (24.9)	55 (31.1)	78 (44.1)	
Physician/APP	8 (2.3)	1 (12.5)	1 (12.5)	6 (75.0)	
Clinical, non-nursing, non-physician	46 (23.8)	19 (22.9)	24 (30.1)	39 (47.0)	
Non-clinical staff	21 (10.9)	8 (21.6)	14 (37.8)	15 (40.5)	
Number of years of experience in current role					0.63
<2 years	46 (13.2)	13 (28.3)	16 (34.8)	17 (37.0)	
2–5 years	70 (20.1)	12 (17.4)	20 (29.0)	37 (53.6)	
6–10 years	50 (14.3)	12 (24.5)	12 (24.5)	25 (51.0)	
>10 years	182 (52.2)	42 (23.9)	53 (30.1)	81 (46.0)	
Number of years of experience in healthcare					0.34
<5 years	38 (10.9)	7 (18.4)	17 (44.7)	14 (36.8)	
5–10 years	55 (15.8)	16 (29.6)	15 (27.8)	23 (42.6)	
11–15 years	47 (13.5)	8 (18.2)	15 (34.1)	21 (47.7)	
16+ years	207 (59.3)	48 (23.7)	54 (26.6)	101 (49.8)	
In leadership or decision-making role in department/division/unit					0.15
No	242 (69.3)	56 (23.7)	77 (32.6)	103 (43.6)	
Yes	101 (28.9)	22 (22.2)	23 (23.2)	54 (54.6)	
Parent or caregiver for a child under the age of 18					0.69
No	191 (54.7)	40 (21.5)	59 (31.7)	87 (46.8)	
Yes	155 (44.4)	38 (25.0)	43 (28.3)	71 (46.7)	
Currently provide, or have provided in the last year, unpaid caregiving help to another adult					0.18
No	201 (57.6)	39 (19.9)	58 (29.6)	99 (50.5)	
Yes	148 (42.4)	40 (27.6)	44 (30.3)	61 (42.1)	

*p-value comes from Fisher exact test.

*Row percentages are rounded and may not total 100. Bold values indicate *p* < 0.05.

In bivariate regressions on eight wellness and workplace outcomes, all except witnessing abuse of power by those in authority showed significant associations with the highest ACEs category—compared to 0 reported ACEs—and/or with the lowest PCEs category, compared to reporting 6–7 PCEs (Table 5). Once both ACEs and PCEs were included in the same model, some of those associations disappeared. Having 4–10 ACEs continued to be a significant predictor of resilience, contributing to a 0.9 (95% CI: 0.00, 1.80) point increase in the total scale score relative to having 0 ACEs, when controlling for PCEs. In comparison, having few PCEs contributed to a 1.61 (95% CI: −2.52, −0.71) decrease in the resilience score relative to 6–7 PCEs, when holding ACEs constant (Table 6). The relationship between having few PCEs and adult outcomes persisted for other variables, even when adjusting for ACEs. Those with the fewest (0–2) PCEs were almost 2.5 times as likely (OR: 2.47, 95% CI 1.13, 5.38) to report compassion fatigue as those with the most (6–7) PCEs; were less likely to report feeling like a valued, equal member of their team whose voice was empowered (OR: 0.31, 95% CI 0.15, 0.63); and were much less likely (OR: 0.23, 95% CI: 0.11, 0.47) to report feeling like they could be autonomous and function to their highest capacity in their workplace. For the outcome of reporting being able to get needed emotional or social support, even having 3–5 PCEs was a predictor of significantly lower odds of responding ‘usually’ or ‘always’ getting such support (OR: 0.32, 95% CI: 0.18, 0.58) compared to those with 6–7 PCEs, while those with 0–2 PCEs (compared to 6–7 PCEs) had 86% lower odds of regular support (OR: 0.14, 95% CI: 0.07, 0.29).

**Table 5:** Bivariate, nested regression of key outcomes dichotomized (clustered on location).

	Burnout in past two weeks *(Yes vs. No [reference])*			Compassion fatigue in past two weeks *(Yes vs. No)*			Frequency of getting needed emotional or social support *(Usually/always vs. Never/rarely/sometimes)*			Resilience score *(continuous)*			Organizational belonging: Feel a valued, equaled member of department/team and voice is empowered *(Very/completely true vs. Not at all/somewhat/moderately true)*			Organizational belonging: Can be autonomous and function to highest capacity *(Very/completely true vs. Not at all/somewhat/moderately true)*			Experienced any workplace violence (physical or non-physical), in past 6 months *(Yes vs. No)*			Experienced any actions by someone in position of authority perceived as abusing their power, in past 6 months *(Yes vs. No)*		
	OR	95% CI	*p*	OR	95% CI	*p*	OR	95% CI	*p*	Coeff.	95% CI	*p*	OR	95% CI	*p*	OR	95% CI	*p*	OR	95% CI	*p*	OR	95% CI	*p*
*Model Covariate*: Adverse Childhood Experiences (ACEs)																								
0 ACEs	ref			ref			ref			ref			ref			ref			ref			ref		
1–3 ACEs	1.5	0.9, 2.5	0.11	0.8	0.4, 1.5	0.48	0.8	0.5, 1.4	0.46	0.3	−0.4, 1.0	0.40	0.9	0.6, 1.5	0.78	1.1	0.6, 1.7	0.84	0.9	0.5, 1.6	0.76	0.8	0.4, 1.6	0.53
4–10 ACEs	1.7	1.0, 3.0	0.05	2.02	1.1, 3.8	**0.03**	0.39	0.2, 0.7	**0.001**	0.1	−0.7, 0.9	0.75	0.7	0.4, 1.2	0.22	0.8	0.4, 1.4	0.37	2.0	1.1, 3.6	**0.02**	1.8	0.9, 3.5	0.09
*Model Covariate*: Positive Childhood Experiences (PCEs)																								
6–7 PCEs	ref			ref			ref			ref			ref			ref			ref			ref		
3–5 PCEs	1.1	0.7, 1.9	0.66	1.2	0.6, 2.3	0.57	0.3	0.2, 0.6	**<0.001**	−0.3	−1.0, 0.4	0.42	0.8	0.5, 1.3	0.29	0.9	0.5, 1.5	0.65	1.2	0.7, 2.0	0.56	1.0	0.5, 2.0	0.97
0–2 PCEs	2.1	1.2, 3.6	**0.01**	2.9	1.5, 5.3	**0.001**	0.1	0.1, 0.3	**<0.001**	−1.2	−1.9, −0.4	**0.002**	0.4	0.2, 0.6	**0.001**	0.3	0.2, 0.5	**<0.001**	2.3	1.3, 4.0	**0.005**	1.9	1.0, 3.7	0.05

*Row percentages are rounded and may not total 100. Bold values indicate *p* < 0.05.

**Table 6:** Multivariate (both ACEs and PCEs in model), nested regression of key outcomes dichotomized (clustered on location).

	Burnout in past 2 weeks *(Yes vs. No [reference])*			Compassion fatigue in past 2 weeks *(Yes vs. No)*			Frequency of getting needed emotional or social support *(Usually/always vs. Never/rarely/sometimes)*			Resilience score *(continuous)*			Organizational belonging: Feel a valued, equaled member of department/team and voice is empowered *(Very/completely true vs. Not at all/somewhat/moderately true)*			Organizational belonging: Can be autonomous and function to highest capacity *(Very/completely true vs. Not at all/somewhat/moderately true)*			Experienced any workplace violence (physical or non-physical), in past 6 months *(Yes vs. No)*			Experienced any actions by someone in position of authority perceived as abusing their power, in past 6 months *(Yes vs. No)*		
	OR	95% CI	*p*	OR	95% CI	*p*	OR	95% CI	*p*	Coeff.	95% CI	*p*	OR	95% CI	*p*	OR	95% CI	*p*	OR	95% CI	*p*	OR	95% CI	*p*
ACEs																								
0	ref			ref			ref			ref			ref			ref			ref			ref		
1–3	1.32	0.78, 2.25	0.31	0.66	0.33, 1.33	0.24	1.27	0.71, 2.26	0.43	0.63	−0.08, 1.35	0.08	1.08	0.64, 1.85	0.77	1.28	0.74, 2.21	0.37	0.79	0.44, 1.41	0.42	0.78	0.38, 1.60	0.49
4–10	1.33	0.69, 2.59	0.39	1.27	0.59, 2.76	0.54	1.08	0.53, 2.18	0.83	0.90	0.00, 1.80	**0.05**	1.27	0.64, 2.51	0.50	1.63	0.81, 3.29	0.17	1.50	0.75, 2.98	0.25	1.42	0.62, 3.23	0.41
PCEs																								
6–7	ref			ref			ref			ref			ref			ref			ref			ref		
3–5	1.01	0.59, 1.74	0.97	1.26	0.63, 2.52	0.52	0.32	0.18, 0.58	**<0.001**	−0.52	−1.25, 0.21	0.16	0.70	0.41, 1.21	0.20	0.77	0.44, 1.34	0.35	1.10	0.61, 1.98	0.75	0.93	0.45, 1.94	0.86
0–2	1.70	0.88, 3.27	0.12	2.47	1.13, 5.38	**0.02**	0.14	0.07, 0.29	**<0.001**	−1.61	−2.52, −0.71	**<0.001**	0.31	0.15, 0.63	**0.001**	0.23	0.11, 0.47	**<0.001**	1.70	0.85, 3.40	0.13	1.49	0.66, 3.38	0.34

*Row percentages are rounded and may not total 100. Bold values indicate *p* < 0.05.

In sub-analyses summarizing distribution of 10 measures of interest for nurses only (Table 7), comparisons between campuses were similar to those for the full study sample. Significant differences between campuses persisted for two variables: getting needed emotional or social support, and resilience. On Campus A, nurses reported usually or always getting needed emotional or social support 69.3% of the time, compared to 52.3% on Campus B, and the average resilience score was 1 point higher on Campus A.

**Table 7:** Distribution of responses for key variables, overall and by site, for nursing respondents only.

	Overall	Campus A	Campus B	*p*-value*
	*n* (%)	*n* (%)	*n* (%)	
Total	184 (100.0)	109 (100.0)	75 (100.0)	
Adverse childhood experiences (ACEs)				0.57
0 ACEs	64 (35.6)	27 (36.5)	37 (34.9)	
1–3 ACEs	66 (36.7)	24 (32.4)	42 (39.6)	
4–10 ACEs	50 (27.8)	23 (31.1)	27 (25.5)	
Positive childhood experiences (PCEs)				0.17
6–7 PCEs	78 (44.1)	29 (38.7)	49 (48.0)	
3–5 PCEs	55 (31.1)	22 (29.3)	33 (32.4)	
0–2 PCEs	44 (24.9)	24 (32.0)	20 (19.6)	
Burnout in past 2 weeks				0.88
No	94 (51.1)	39 (52.0)	55 (50.5)	
Yes	90 (48.9)	36 (48.0)	54 (49.5)	
Compassion fatigue in past 2 weeks				1.00
No	139 (75.5)	57 (76.0)	82 (75.2)	
Yes	45 (24.5)	18 (24.0)	27 (24.8)	
Frequency of getting needed emotional or social support				**0.02**
Never/rarely/sometimes	75 (40.8)	23 (30.7)	52 (47.7)	
Usually/always	109 (59.2)	52 (69.3)	57 (52.3)	
Resilience, *n* (mean, SE)	179 (14.3, 0.2)	72 (14.9, 0.3)	107 (13.9, 0.29)	**0.02**
Organizational belonging: Feel a valued, equaled member of department/team and voice is empowered				
Not at all/somewhat/moderately true	104 (56.5)	36 (48.0)	68 (62.4)	0.07
Very/completely true	80 (43.5)	39 (52.0)	41 (37.6)	
Organizational belonging: Can be autonomous and function to highest capacity				0.29
Not at all/somewhat/moderately true	86 (47.0)	31 (41.9)	55 (50.5)	
Very/completely true	97 (53.0)	43 (58.1)	54 (49.5)	
Experienced any workplace violence (physical or non-physical), past 6 months				0.88
No	112 (60.9)	45 (60.0)	67 (61.5)	
Yes	72 (39.1)	30 (40.0)	42 (38.5)	
Experienced any actions by someone in position of authority perceived as abusing their power, past 6 months				0.56
No	150 (81.5)	63 (84.0)	87 (79.8)	
Yes	34 (18.5)	12 (16.0)	22 (20.2)	

*p-value comes from Fisher exact test.

**p-value comes from two-sided, two sample test of means (t-test in Stata).

*Row percentages are rounded and may not total 100. Bold values indicate *p* < 0.05.

In hierarchical regression, the addition of dichotomous teammate role as a covariate into the model did not meaningfully change associations between ACEs, PCEs and the eight outcomes of interest. In the resilience model, having 4–10 ACEs was still associated with an increase in resilience score compared to 0 ACEs, while having 0–2 PCEs was still associated with a decreased resilience score compared to having 6–7 PCEs, and being a nurse was associated with a decrease of 0.70 points (*p* = 0.03) in resilience compared to all other roles. For the outcome of any WPV in the preceding 6 months, neither ACEs nor PCEs were associated with the outcome, but being a nurse was associated with almost double the odds (OR = 1.93, *p* = 0.008) of having experienced violence relative to all other role categories combined (Table 8).

**Table 8:** Multivariate (ACEs, PCEs, dichotomized role), nested regression of key outcomes dichotomized (clustered on location).

	Burnout in past 2 weeks *(Yes vs. No [reference])*			Compassion fatigue in past 2 weeks *(Yes vs. No)*			Frequency of getting needed emotional or social support *(Usually/always vs. Never/rarely/sometimes)*			Resilience score *(continuous)*			Organizational belonging: Feel a valued, equaled member of department/team and voice is empowered *(Very/completely true vs. Not at all/somewhat/moderately true)*			Organizational belonging: Can be autonomous and function to highest capacity *(Very/completely true vs. Not at all/somewhat/moderately true)*			Experienced any workplace violence (physical or non-physical), in past 6 months *(Yes vs. No)*			Experienced any actions by someone in position of authority perceived as abusing their power, in past 6 months *(Yes vs. No)*		
	OR	95% CI	*p*	OR	95% CI	*p*	OR	95% CI	*p*	Coeff.	95% CI	*p*	OR	95% CI	*p*	OR	95% CI	*p*	OR	95% CI	*p*	OR	95% CI	*p*
ACEs																								
0	ref			ref			ref			ref			ref			ref			ref			ref		
1–3	1.37	0.80, 2.35	0.26	0.65	0.32, 1.31	0.23	1.25	0.70, 2.23	0.45	0.63	−0.10, 1.37	0.09	0.98	0.58, 1.67	0.95	1.20	0.69, 2.08	0.52	0.75	0.41, 1.36	0.34	0.81	0.39, 1.67	0.56
4–10	1.34	0.68, 2.62	0.40	1.25	0.57, 2.71	0.58	1.00	0.50, 2.02	1.00	0.96	0.04, 1.89	**0.04**	1.13	0.57, 2.23	0.73	1.54	0.76, 3.12	0.23	1.30	0.64, 2.64	0.46	1.48	0.64, 3.39	0.36
PCEs																								
6–7	ref			ref			ref			ref			ref			ref			ref			ref		
3–5	1.03	0.60, 1.78	0.97	1.24	0.62, 2.48	0.54	0.33	0.19, 0.59	**<0.001**	−0.44	−1.18, 0.31	0.25	0.71	0.41, 1.21	0.21	0.75	0.43, 1.31	0.31	1.05	0.58, 1.91	0.88	0.96	0.46, 1.99	0.91
0–2	1.69	0.87, 3.29	0.12	2.38	1.09, 5.20	**0.03**	0.17	0.08, 0.34	**<0.001**	−1.49	−2.41, −0.57	**0.002**	0.37	0.18, 0.74	**0.005**	0.24	0.12, 0.48	**<0.001**	1.75	0.86, 3.53	0.12	1.47	0.65, 3.34	0.36
Role																								
Other	ref			ref			ref			ref			ref			ref			ref			ref		
Nurse	1.53	0.97, 2.39	0.07	1.18	0.69, 2.03	0.54	1.42	0.89, 2.28	0.15	−0.70	−1.31, −0.09	**0.03**	1.00	0.64, 1.57	0.99	0.92	0.58, 1.46	0.72	1.93	1.19, 3.13	**0.008**	0.95	0.54, 1.69	0.87

*Row percentages are rounded and may not total 100. Bold values indicate *p* < 0.05.

## Discussion

This paper summarized study findings from survey data on childhood and adult experiences, as well as the growth of trauma-informed care training development and implementation efforts in an academic health system across a 10-year period that set the stage for conducting the research. Data were collected from interdisciplinary, clinical and non-clinical participants across two hospital campuses, as part of the leadup to campus-wide trauma-informed care training, with additional analyses focusing specifically on the experiences of nurses. Study findings highlight ACEs and PCEs among healthcare employees and are uniquely able to explore the extent to which these were predictors of adult wellness and workplace outcomes.

Among our sample of 349 participants, numbers of ACEs reported were higher than for population data. Hege et al. (19) analyzed data from the 2012 NC BRFSS and found that nearly 60% of participants reported at least one ACE and 16.6% reported four or more ACEs. For comparison, among our respondents, 61.1% had at least one reported ACE, but 24.9% had four or more. These findings build upon some previously available ACEs data among clinician populations, including one study which found a much larger proportion of participants who reported 4+ ACEs among a national sample of nursing students compared to the national average (8). They also highlight that ACEs are not only common among the general population and ergo patients but are also common among the health system workforce. This combination can greatly impact the wellbeing of patients, teammates, organization, and communities. Our data also indicate that younger and/or less experienced healthcare professionals appeared to report higher numbers of ACEs than more experienced counterparts. It is possible this is due to a combination of greater training and education about trauma-informed care being increasingly delivered in clinical learner programs and thus may have contributed to more awareness of what are ACEs or less stigma associated with reporting them. However, it also points to the importance of providing more training and resources to incoming teammates who may still be learning how to navigate difficult workplaces and patient interactions.

This study also contributes to recent growing evidence that describes the role of PCEs in future wellbeing irrespective of ACEs, and that research and health promotion interventions should consider both ACEs and PCEs (4, 5). This was exhibited in our data, which showed that in adjusted models including both variables, only a relationship between ACEs and resilience remained (with more ACEs unexpectedly predicting higher resilience) in comparison to associations between PCEs and key adult outcomes (compassion fatigue, organizational belonging, and getting need emotional or social support) that persisted. The unexpected finding between ACEs and resilience may in part reflect that while having more ACEs has been shown in other literature to predict challenges with future employment (20, 21), as Maunder and colleagues comment: “it is possible that healthcare workers who have experienced abuse represent a biased sample which is skewed toward resilient outcomes of early abuse” (2020, p. 120). Nevertheless, recognizing that people with fewer childhood experiences of being supported by and connected to family, friends, and community reported greater difficulties in accessing support in adulthood or felt a lower sense of organizational belonging helps link back to the value of trauma-informed care principles and possible opportunities for intervention to foster empowerment and intentional inclusion of all voices into healthcare.

In collecting campus-wide data from two health system campuses, we were also able to capture some variations in key outcomes between these locations. Namely, participants on one campus (Campus A—suburban location) reported being significantly more likely to get their needed emotional or social support, to have higher resilience, and to feel like valued and empowered members of their teams. These may reflect differences in employee populations more broadly and/or may reflect differences in the two campus environments and histories of the hospitals that may foster a greater sense of teammate support in one than the other. Underlying disparities in population-level mental health between more urban and rural areas, such as in accessing skilled treatment and resources, may also contribute to differences observed between the campuses (22). Nevertheless, sizable proportions of individuals on both campuses reported challenges with accessing support and other indicators of wellbeing and connected, including in one setting that has a stronger reputation of being a campus “family.” Further, it gives evidence to the importance of conducting organizational assessments and developing tailored interventions that meet unique needs of local campuses and communities as opposed to one-size-fits-all solutions implemented across health systems more broadly.

Moreover, nurses on average reported lower resilience compared to all other participant roles combined, also pointing to the importance of understanding and responding to unique needs of groups within the health system. This further makes the case for advancing trauma-informed care initiatives as they can also provide tools to manage personal wellbeing (23) and build resilience, which is a key mechanism for improving health and reducing the perpetuation of traumatic and adverse experiences in adulthood and in future generations (24). Some of these adverse experiences in adulthood can include WPV, which nurses too reported significantly higher rates of compared to all other participants combined. Among our overall campus populations about one-third of respondents reported WPV in the past 6 months, as did almost 40% of nurses. For comparison, one study found 76% of nurses in one system reported WPV in the past year (25) which is a longer time frame than we asked participants about, while 88% of respondents in an emergency department reported any violence in 6 months (18), reflecting a higher stress context. Trauma-informed care also offers a framework for teaching teammates skills for responding to conflict and activated patients and visitors—and for understanding how prior trauma may contribute to individuals’ stress behaviors—and for in turn reducing WPV.

### Limitations

There are several limitations to consider to these findings. Our study was conducted on two smaller campuses (one suburban and one rural) in a larger academic healthcare system, so workplace dynamics or participant experiences may look different on bigger campuses and/or in locations based in dense urban areas. We also deliberately did not collect participant demographic data as a way to encourage greater participation and to increase openness of responding, so we are limited in conclusions we can draw about the relationship between individual characteristics and adult outcomes, as well as the ability to characterize the personal demographics of our sample. Future studies should aspire to include such variables in data collected. Furthermore, there are gaps in roles represented in the data (e.g., physicians and APPs were largely missing) that limit generalizability to other health contexts and across disciplines. The survey was also recruited in concert with advertisement for trauma-informed care training in a workplace setting, which may have contributed to bias in self-selection to participate by those who strongly resonated with the importance of trauma-informed care, who felt enough trust and commitment to the institution to be willing to contribute their time, and or to those whose leaders otherwise encouraged participation as important to the institution’s mission and promoted taking time to complete the survey and training (which was particularly true for nurses). However, we believe that the alignment between the training opportunities and the survey contributed to higher participation and greater representation of the employee populations on both campuses than if we had tried to recruit for the survey independently.

### Lessons learned, and implications for practice and nursing

Reaching a point where hospital leaders were willing to support and endorse concurrent delivery of campus-wide trauma-informed education and survey participation to assess related topics was a process demanding great patience. Elements that helped make this possible and enabled more compelling communication included having a recognized trauma-informed care expert, who was a nurse; collaboration with an interdisciplinary team; tailoring content to deliver data and information in the most impactful way to various audiences; and steadily growing awareness of the long-term impacts of trauma and support for the concept of trauma-informed care throughout the system.

Our findings of commonly reported adversities among teammates and particularly how fewer PCEs contribute to worse outcomes in adulthood, help augment the argument for health systems to evolve and recognize challenges faced not only by patients but also by their workforce. Nevertheless, we have witnessed many structural barriers that can stand athwart broad adoption of changes that might contribute to improved resilience, providing greater support, and engaging teammates in a way that promotes feelings and experiences of organizational belonging. First and foremost, healthcare systems often have operational histories grounded in hierarchical and patriarchal processes, with internal cliques and rivalries between disciplines, discipline specific jargon and workflows, and operational and departmental silos. These challenges foster power dynamics and encourage political agendas that can fuel mistrust, reduce safety, gender inequity, cultural differences, reduced diversity and inclusion, and decreased collaboration, empowerment, voice, and choice. Such historical structures do not support an environment where trauma-informed care can naturally occur or flourish, and unfortunately, may exacerbate trauma experiences of healthcare workers.

Second, healthcare commonly acts in a short-term and reactive manner and expects quick action and outcomes, while adopting a trauma-informed approach requires a longer-term focus to promote widespread, sustainable change. (See Supplementary material for examples of how our team worked to embed trauma-informed principles across domains, and our core tenets and recommendations with a longer-term perspective.) A final challenge is the tension between scaling changes throughout larger systems to reach more people, while maintaining fidelity to the key principles and components of trauma-informed initiatives, and ensuring that the work is still locally relevant. We contend though that not only are nurses important targets for trauma-informed initiatives such as for strengthening resilience and providing support following experiences of WPV, they are well-positioned to help lead widespread change and help overcome the challenges discussed.

Nurses are ubiquitous throughout the system and function in varying roles from students, bedside staff, to chief nursing officers over multiple campuses. Furthermore, nurses are vital in interdisciplinary teams that work to promote health and cultivating change for patients, teammates, and organizations (26, 27). There are opportunities to embed skills and support-building and trauma-informed practices into nursing classroom curriculums, clinical rotations, competencies, orientation and annual training, nurse leadership seminars and retreats, and continuing education, in order to reach a sizable proportion of the healthcare system. Importantly, nurses have a “front row seat” to witnessing how trauma, adversity, and daily difficulties affect teammates and patients alike and are thus critical voices in shaping strategies for intervention and stakeholders for implementing them; indeed, nurse leadership set the foundation for and propelled the work presented in this paper.

Our study provides data to be used to guide future efforts that are responsive to local need, while also highlighting the importance of recognizing the absence of PCEs as a key type of adversity with long-term implications on wellbeing in the healthcare workforce. Nursing is a key profession to facilitate system change for health promotion to foster greater support for and engagement of all teammates in the healthcare system, and for elevating our understanding of “do no harm” to patients and each other.

## Data Availability

The raw data supporting the conclusions of this article will be made available by the authors without undue reservation.
