# Supplementary material for: Negative effects of adverse childhood experiences and absence of positive childhood experiences on healthcare employees: survey findings built on 10 years of trauma-informed development
Source: Front Public Health. 2025 Jan 6;12:1494587. doi: 10.3389/fpubh.2024.1494587 (PMC11743665; doi:10.3389/fpubh.2024.1494587)
Supplement: Supplementary file 1 [file Data_Sheet_1.docx]

**Supplementary Materials**

Pursuing Trauma-Informed Care in an Academic Health Care Setting

Efforts to promote adoption of the trauma-informed care had been built around a foundation of delivering training as a preliminary step for building awareness of trauma and adversity, their sequelae, and how healthcare systems and their workforce can change their practices to develop a trauma-informed setting. Below are materials that illustrate implementation of this work leading up to the study. **Supplementary Figure 1** lists topics areas introduced in the foundational training (delivered concurrent with data collection in this study) and explored in greater detail by those who sought out further training. **Supplementary Figure 2** identifies training audiences and training focal areas that were successfully reached in and adjacent to our system. **Supplementary Figure 3** defines the 10 organizational domains for trauma-informed practices named by the SAMHSA Framework and provides examples of local health system activities the co-authors identified or conceptualized could be applied to the 10 domains. Finally, we summarize our organizational vision for guiding trauma-informed change in **Supplementary Figure 4**, naming five fundamentals and aspirations for healthcare systems and their leaders.

**Supplementary Figure 1.** Trauma-Informed Care Training Content Areas


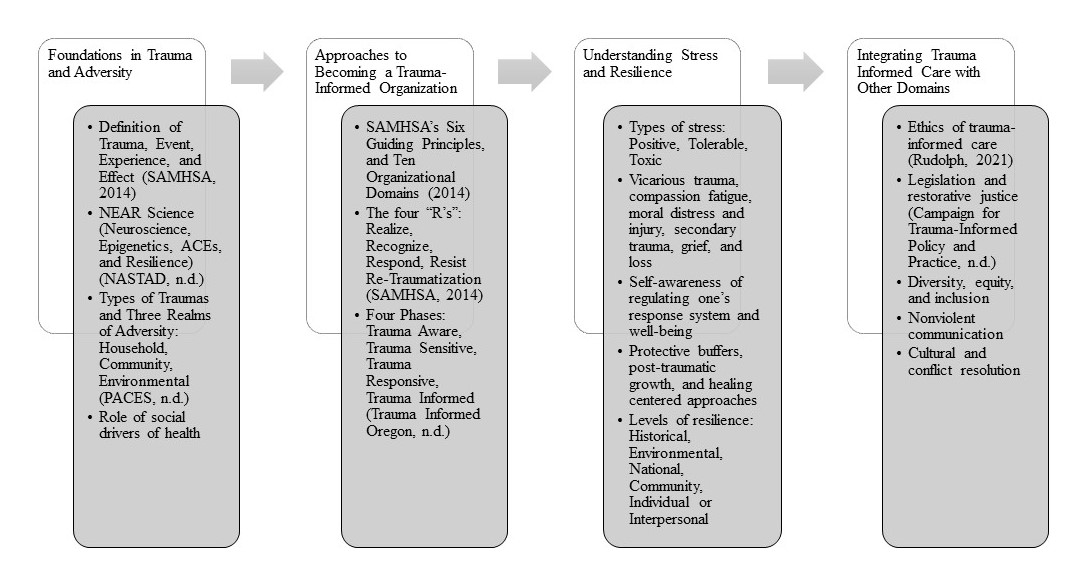


**Supplementary Figure 2.** Historical Training Audiences and Tailored Training Content


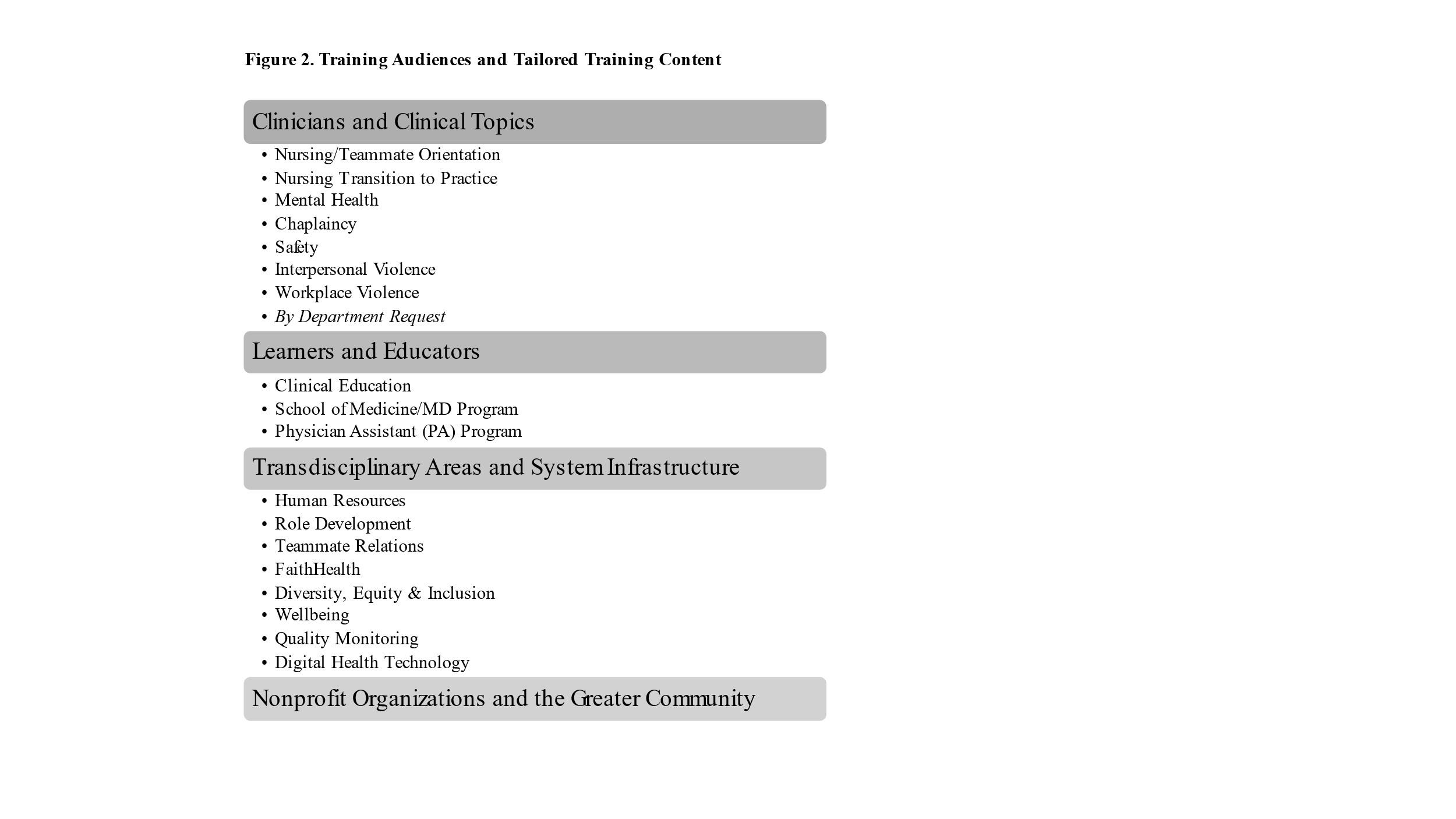


**Supplementary Figure 3.** 10 Organizational Domains (SAMHSA, 2014) with Definitions and Example Health System Activities


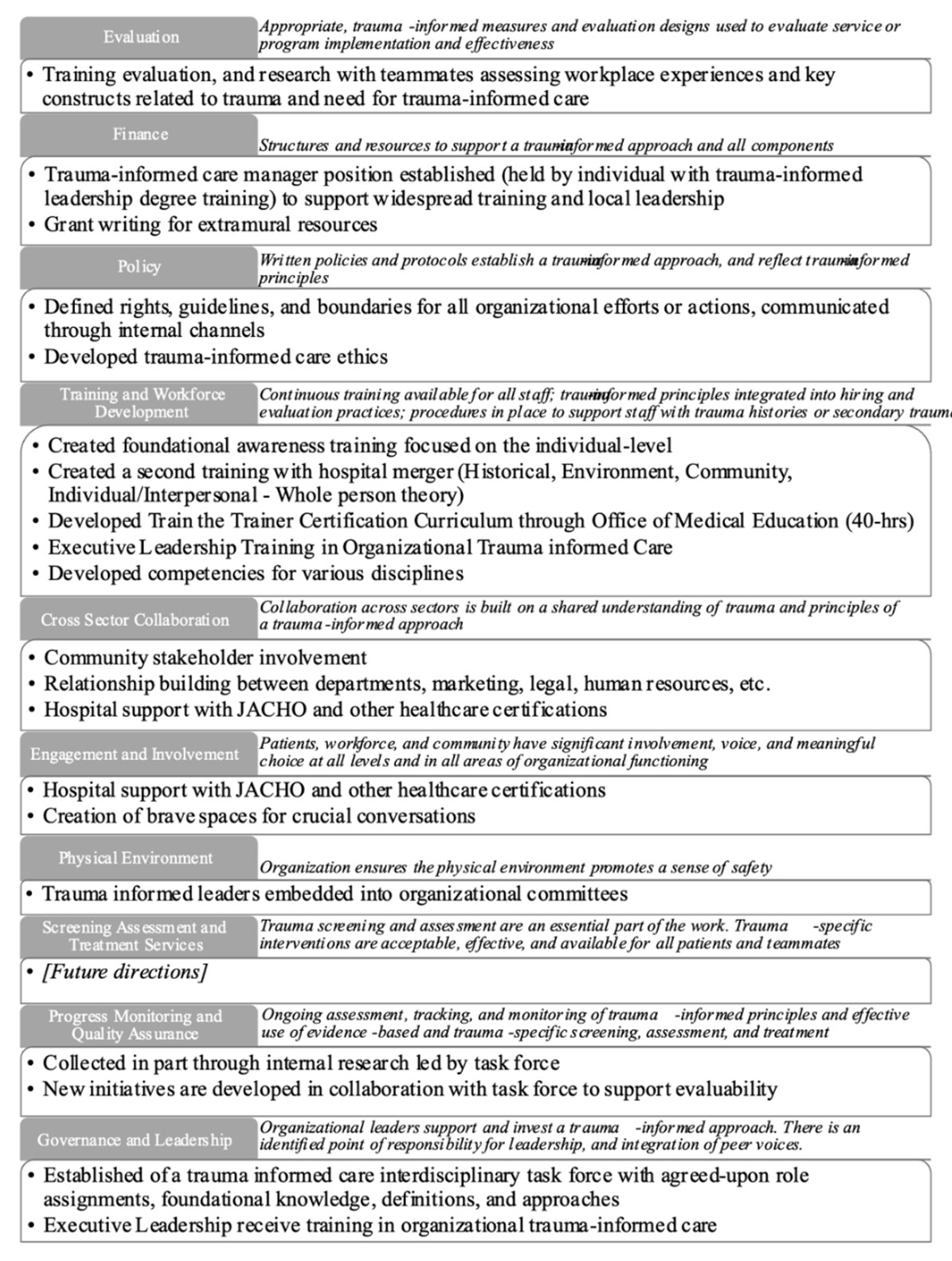


**Supplementary Figure 4.** Organizational Vision for Trauma-Informed Health Promotion

**References for Supplementary Materials**

CTIPP: Campaign for Trauma-Informed Policy and Practice (n.d.). Policy. www.ctipp.org/policy [Accessed September 10, 2024]

Hormann, S., & Vivian, P. (2017). *Intervening in Organizational Trauma: A Tale of Three Organizations* (pp. 175–189). https://doi.org/10.1007/978-3-319-47045-0_11

NASTAD. (n.d.). Trauma-informed approaches toolkit: NEAR science. https://nastad.org/trauma-informed-approaches-toolkit/near-science [Accessed September 9, 2024]

PACEsConnection. (n.d.). 3 Realms of ACEs. https://www.pacesconnection.com/pages/3RealmsACEs [Accessed September 9, 2024]

Rudolph K. (2021). Ethical Considerations in Trauma-Informed Care. The Psychiatric clinics of North America, 44:4, 521–535. doi: 10.1016/j.psc.2021.07.001

Substance Abuse and Mental Health Services Administration. (2014). *SAMHSA’s Concept of Trauma and Guidance for a Trauma-Informed Approach* (Issue HHS Publication No. (SMA) 14-4884). https://ncsacw.acf.hhs.gov/userfiles/files/SAMHSA_Trauma.pdf [Accessed September 9, 2024]

Trauma Informed Oregon. (n.d.) Roadmap to Trauma Informed Care. https://traumainformedoregon.org/implementation/implementation-and-accountability-overview/roadmap-to-trauma-informed-care/ [Accessed September 10, 2024]
